# Addition of Section 10, Rules 66–73 for further integration of Candidatus names into the International Code of Nomenclature of Prokaryotes

**DOI:** 10.1099/ijsem.0.006638

**Published:** 2025-01-08

**Authors:** Aharon Oren

**Affiliations:** 1 Institute of Life Sciences, The Hebrew University of Jerusalem, The Edmond J. Safra Campus, 9190401 Jerusalem, Israel

**Keywords:** *Candidatus*, emendation, International Code of Nomenclature of Prokaryotes, International Committee on Systematics of Prokaryotes, pro-valid publication

## Abstract

Following a proposal for further integration of *Candidatus* names into the International Code of Nomenclature of Prokaryotes, I here report the outcome of the ballot on this proposal by the members of the International Committee on Systematics of Prokaryotes.

## Introduction

In January 2024, D.R. Arahal *et al.* [1] published a proposal for further integration of *Candidatus* names in the International Code of Nomenclature of Prokaryotes [2].

A public discussion of the proposal was held between January and July 2024 [see Article 13 (b)(4) and Article 4(d) of the Statutes of the International Committee on Systematics of Prokaryotes (ICSP) [3]]. The collated comments can be found in the attached supplementary information. In response to a comment raised, the authors presented a modified version of the proposal.

The members of the ICSP voted on this proposal in September–December 2024. C.M. Manaia (Vice-Chair, ICSP) and A. Oren (Executive Secretary, ICSP) collated the results of the vote. Out of the 53 members of the ICSP with voting rights, 36 endorsed the emendations proposed by Arahal *et al.*, 8 voted against and there was 1 abstention. Based on these votes, the following Section 10 containing Rules 66–73 was approved:

## Section 10. *Candidatus* names

### Rule 66

The *Candidatus* status should be used to propose the names of prokaryotic taxa for which some information is available (Rules 67 and 68), but for which the requirements for valid publication of a name under Rule 27(3) are not met.

(1) A *Candidatus* name consists of the word ‘*Candidatus*’, followed by a taxon name formed in accordance with Sections 2 and 3 of this *Code*.

(2) *Candidatus* names are not validly published and cannot be the correct name of a taxon. However, *Candidatus* names are regulated by this *Code* analogously to validly published names. Unless otherwise indicated, terms specific to the regulation of *Candidatus* names are derived from the analogous terms used for the regulation of names in other sections of this *Code* by adding the prefix *pro-*. *Candidatus* names have a nomenclatural pro-status and may have pro-standing in nomenclature (Rules 67 and 68), the main consequences of which are implemented by Rules 71–73.

(3) *Candidatus* names may be pro-validly published – the name is not validly published but is included in an effective publication (Rules 25a and 25b) and meets certain other requirements (Rules 67 and 68); pro-legitimate – the name is pro-validly published and would be legitimate (Section 8) if it was validly published; pro-illegitimate – pro-validly published and not pro-legitimate; pro-correct – the name that must be adopted for a taxon under the Rules if the taxon does not have a correct name and it is of interest to assign a name to it (Rule 71).

(4) A pro-validly published and pro-legitimate *Candidatus* name cannot compete with a validly published and legitimate name for priority (Rule 23a), but it can compete with another pro-validly published and pro-legitimate *Candidatus* name for pro-priority (Rule 71), by analogy with Rule 23a.

(5) If a nomenclatural question arises in connection with a *Candidatus* name, which is not expressly dealt with in Section 10, the solution to be chosen is in accordance with the Rules and has the most obvious analogy to a nomenclatural solution provided for in other Sections of this *Code*. Doubtful cases should be referred to the Judicial Commission (Appendix 8). The replacement of a pro-illegitimate name or epithet is conducted by analogy with Rules 54 and 55, but see also Rule 72(4)(b).

*Note*. For the history of the *Candidatus* status, see Appendix 11. Section 10 supersedes Appendix 11 in the event of a conflict.

## Pro-valid publication of a Candidatus name

### Rule 67

A *Candidatus* name for a new taxon, or a new combination or *nomen novum* for an existing *Candidatus* taxon, is not pro-validly published unless the following criteria are met:

(1) The name meets the requirements for publication in Rule 27(1).

(2) The name meets the requirements for the formation of *Candidatus* names in Rule 66(1) in conjunction with the requirements for taxon descriptions in Rule 27(2).

(3) The name meets the requirements for nomenclatural types in Rule 27(3), except for the following:

(a) In the case of the name of a species or subspecies, the name does not meet the requirements of Rule 30 but meets the requirements of Rule 69.

(b) In the case of the name of a taxon above the rank of species, the name of its nomenclatural type is not validly published, but it is pro-validly published and pro-legitimate.

*Note*. Possible exceptions to Rule 67 are defined in Rule 68(2), Rule 68(3) and Rule 68(4).

### Rule 68

The date of pro-valid publication of a *Candidatus* name is the date of its publication in the *IJSEM*.

(1) If the original proposal of the new *Candidatus* name or new *Candidatus* combination was not published in the *IJSEM*, pro-valid publication of the name may be achieved by its announcement in an *IJSEM Candidatus* List, by analogy with the announcement of a name in a Validation List for the purpose of its valid publication (Rule 27 Note 1).

(2) If a *Candidatus* name does not meet the requirements of Rule 67 but has been included in an *IJSEM Candidatus* List that was published before 1 January 2025, the name is pro-validly published.

(a) If the nomenclatural type of such a name was not mentioned in the *Candidatus* List, a nomenclatural type will be provided in an addendum to the respective *Candidatus* List, published separately in the *IJSEM* by the List Editors. Such an addendum does not affect the date of pro-valid publication. If several possible nomenclatural types in accordance with Rule 67(3) have been provided in the effective publication, one shall be selected.

(b) If a nomenclatural type in accordance with Rule 67(3) cannot be determined for a name that is pro-validly published because of its inclusion in a *Candidatus* List, this shall also be announced in an addendum to a *Candidatus* List, and the name considered pro-illegitimate.

(3) If a name meets the requirements given in Rule 67, with the exception of the use of the word ‘*Candidatus*’ as stipulated by Rule 67(2), and the name is included in an *IJSEM Candidatus* List, then the name is pro-validly published and is considered to be a *Candidatus* name.

(4) When a name that has been considered validly published is found to not meet the requirements for nomenclatural types in Rule 27(3) but is found to meet the requirements of Rule 67(3), the name should be given *Candidatus* status by announcement in an *IJSEM Candidatus* List, stating the reasons for not meeting the requirements of Rule 27(3) and including the information required by Rule 67(3). The presumed date of valid publication of the name shall then become its date of pro-valid publication.

(5) A *Candidatus* name that has previously been pro-validly published in the *IJSEM* may be included in an *IJSEM Candidatus* List for the sole purpose of its orthographic or grammatical correction (Section 9). Such inclusion shall not affect the date of pro-valid publication.

*Note*. In order to determine the relative pro-priority of *Candidatus* names that were included in the same *Candidatus* List for the purpose of their pro-valid publication, each included name should be assigned a number reflecting the date of receipt of the request for inclusion in the *Candidatus* List, by analogy with Rule 24b(4). Within a *Candidatus* List that does not contain such numbers, the relative pro-priority of each name is determined by the date of its original publication as indicated in the *Candidatus* List. *Candidatus* Lists published prior to 1 January 2025 may indicate more than one original publication per name. If so, the relative pro-priority of a name is determined by the date of the earliest indicated original publication of that name.

## Nomenclatural type of a *Candidatus* name

### Rule 69

The nomenclatural type of a pro-validly published *Candidatus* name is regulated by analogy with Section 4, except for the following:

(1) The kind of nomenclatural type of a pro-validly published *Candidatus* name at the rank of species or subspecies is one of the following (in order of decreasing preference for the kind of nomenclatural type):

(a) A culture containing living cells of the species or subspecies from which characters of use in taxonomy can be obtained, but which does not meet the requirements of Rule 30.

(b) A preserved specimen containing cells of the species or subspecies from which characters of use in taxonomy can be obtained.

(c) A genome sequence obtained from the species or subspecies and deposited in one of the databases belonging to the International Nucleotide Sequence Database Collaboration (INSDC).

(d) A gene sequence obtained from the species or subspecies and deposited in one of the databases belonging to the INSDC.

(2) For the purpose of designating a nomenclatural type under Rule 69(1)(a) or Rule 69(1)(b), the culture or preserved specimen must be deposited in an appropriate collection. Evidence of its availability from that collection must be provided at the time of publication in the *IJSEM*.

(3) For the purpose of designating a nomenclatural type according to Rule 69(1)(c) or according to Rule 69(1)(d), its INSDC nucleotide sequence accession numbers must be provided at the time of publication in the *IJSEM*, i.e. identifiers that can be used directly to obtain a nucleotide sequence from INSDC, without the need for an intermediate identifier. The INSDC nucleotide sequence accession numbers provided must specifically and completely cover the nucleotide sequence, and all associated sequence data must be available at the time of publication in the *IJSEM*. All sequence identifiers required to specifically and completely cover the sequence must be cited in the effective publication.

(a) If sequence identifiers other than INSDC nucleotide sequence accession numbers have been cited in the effective publication, INSDC nucleotide sequence accession numbers that can be unambiguously mapped to those identifiers must be provided when requesting the inclusion of the taxon name in an *IJSEM Candidatus* List.

(b) If INSDC nucleotide sequence accession numbers have been cited in the effective publication but not in the protologue (see Rule 27), these INSDC nucleotide sequence accession numbers must be provided when requesting the inclusion of the taxon name in an *IJSEM Candidatus* List.

(4) For the purpose of designating a nomenclatural type under Rule 69(1)(d), the protologue must include a statement indicating that, in the taxonomic opinion of the authors, the gene sequence is sufficiently specific to distinguish the taxon from other taxa of the same rank. If such a statement has not been included in the protologue (see Rule 27), the statement must be provided when requesting the inclusion of the taxon name in an *IJSEM Candidatus* List.

*Note*. Possible exceptions to Rule 69(2), Rule 69(3) and Rule 69(4) are defined in Rule 68(2). The nomenclatural type should conform to minimal standards when these are available for the respective kind of nomenclatural type at the time of effective publication.

## Rule 70

The nomenclatural type of a pro-validly published *Candidatus* name at the rank of species or subspecies may be replaced by another nomenclatural type, provided that the replacement type meets the requirements of Rule 69. Such a proposed replacement must be published in the *IJSEM*.

(1) The possible reasons for replacing the nomenclatural type of a *Candidatus* species or subspecies are the following:

(a) The nomenclatural type is no longer available. This includes deposits of living cultures or specimens that are no longer available and sequence records that have been suppressed by INSDC.

(b) The kind of the proposed replacement type is preferred over the kind of the nomenclatural type, as indicated in Rule 69(1).

(c) The proposed replacement type is of the same kind as the designated nomenclatural type, but the proposed replacement type is of higher quality.

(2) The proposal of a replacement type in the *IJSEM* must state the reason for the replacement in sufficient detail. The proposal must also provide evidence that the replacement type belongs to the species or subspecies according to the taxonomic view expressed in the effective publication of the name of the species or subspecies.

(3) The proposal of a replacement type is void if it does not meet the requirements set out in this Rule. Doubtful cases should be referred to the Judicial Commission (Appendix 8).

*Note*. A replacement type must not be proposed if it meets the requirements of Rule 30. If it does, it should be used instead to propose the name of the species or subspecies for valid publication, provided that this can be done in accordance with Section 5 and Rule 72 of this *Code*.

## Candidatus names with nomenclatural pro-standing

### Rule 71

If a taxon with a given circumscription, position and rank has no correct name (Rule 23a), the taxon can bear only one pro-correct name, i.e. the pro-validly published and pro-legitimate name with the earliest date of pro-valid publication (Rule 68). Exceptions to pro-priority can be made by conservation or rejection.

(1) A pro-validly published name or epithet cannot be conserved over a validly published name or epithet, but a pro-validly published name or epithet may be conserved by the Judicial Commission over another pro-validly published name or epithet by analogy with Rule 56b.

(2) The Judicial Commission may, by analogy with Rule 56a, place a pro-validly published name or epithet on the list of rejected names or epithets. Such rejection equally applies to validly published names or epithets.

*Note*. A taxon for which there is neither a validly published and legitimate name nor a pro-validly published and pro-legitimate name has neither a correct name nor a pro-correct name.

### Rule 72

From 1 January 2025, when proposing a taxon name for the purpose of its valid publication under this *Code* (Rule 27), a pro-validly published and pro-legitimate *Candidatus* name, or epithet thereof, must be reused under certain conditions, depending on the taxonomic view expressed by the authors in the effective publication (Rules 25a and 25b) containing the proposal. Under all other conditions, a pro-validly published and pro-legitimate *Candidatus* name, or epithet thereof, must not be reused. This Rule is not retroactive.

(1) If a name above the rank of species is proposed for the purpose of its valid publication under this *Code*, and if, in the taxonomic opinion of the authors, the proposed name is a synonym of a pro-validly published and pro-legitimate *Candidatus* name of the same rank, then that *Candidatus* name must be reused, unless to do so would contravene a Rule of this *Code*.

(2) If a name at the rank of species or subspecies is proposed for the purpose of its valid publication under this *Code*, and if, in the taxonomic opinion of the authors, the proposed name is a synonym of a pro-validly published and pro-legitimate *Candidatus* name at the same rank and in the same position, then that *Candidatus* name must be reused, unless to do so would contravene a Rule of this *Code*.

(3) If a name at the rank of species or subspecies is proposed for the purpose of its valid publication under this *Code*, and if, in the taxonomic opinion of the authors, the proposed name is a synonym of a pro-validly published and pro-legitimate *Candidatus* name at the rank of species or subspecies and in a different position, then the final epithet of that *Candidatus* name must be reused, unless to do so would contravene a Rule of this *Code*.

(4) Not to reuse a pro-validly published and pro-legitimate *Candidatus* name above genus rank when proposing a name for valid publication, while considering the two names to be synonymous, is permitted where this is a necessary condition to form a name, according to the Rules of this *Code*.

Example. If the hypothetical genus with the pro-validly published and pro-legitimate name ‘*Candidatus* Dedyshiibacter’ was the nomenclatural type of a family with the pro-validly published and pro-legitimate name, ‘*Candidatus* Dedyshiibacteraceae’, and if authors intended to validly publish the name of another genus in the same family, e.g. *Dedyshiibacteroides*, as well as the name of the family, but cannot propose the name *Dedyshiibacter* for valid publication, at the time, then they may propose the name *Dedyshiibacteroidaceae* for the family, but not *Dedyshiibacteraceae*. In this case, the authors should immediately list ‘*Candidatus* Dedyshiibacteraceae’ as a synonym of *Dedyshiibacteroidaceae*.

### Rule 73

The original authors of a *Candidatus* name or epithet reused under Rule 72, whether or not as a synonym, are cited by giving the name of the taxon, followed in parentheses by the word ‘*ex*’ and the name of the original authors and the year of pro-valid publication (see Rule 14b and Rule 33c Note 2). Where other authors are subsequently to be cited in parentheses (Rule 34b), this form of citation is retained by analogy with the perpetuation of ‘*ex*’ in Rule 33c Note 2. Rule 73 is retroactive, but failure to comply with it does not alter the nomenclatural status of a name.

*Note*. The original *Candidatus* name from which a reused epithet is taken is known as the pro-basonym, not the basonym (Rule 34a).

## Supplementary material

10.1099/ijsem.0.006638 Uncited Supplementary Material 1.

## Abbreviations

ICSP: International Committee on Systematics of Prokaryotes
INSDC: International Nucleotide Sequence Database Collaboration
