# Supplementary material for: Addition of Section 10, Rules 66–73 for further integration of Candidatus names into the International Code of Nomenclature of Prokaryotes
Source: Int J Syst Evol Microbiol. 2025 Jan 8;75(1):006638. doi: 10.1099/ijsem.0.006638 (PMC12281813; doi:10.1099/ijsem.0.006638)
Supplement: Uncited Supplementary Material 1. [file ijsem-75-06638-s001.pdf]

21 April – Phil Hugenholtz

Hi All, just adding the link to the relevant paper for this discussion - https://www.microbiologyresearch.org/content/journal/ijsem/10.1099/ijsem.0.006188

1 May – Celia Manaia

The proposed new section of the ICNP seems to be an important step forward to add value to taxonomic studies in which non-culturable prokaryotes are characterised and named. In the section "CONCLUSION: THE PROPOSED NEW SECTION OF THE ICNP" it was not clear to me which type materials will be accepted and how their availability and authenticity will be verified.

1 May – Markus Göker

Celia, thanks for your comment. The information you are asking for is found in the section "Nomenclatural type of a Candidatus name".

17 May – Wee Fei Aaron Teo

Dear esteemed colleagues,I trust this message finds you well.

As part of our ongoing commitment to fostering knowledge exchange and collaboration within the microbiology community, the Bergey’s International Society for Microbial Systematics (BISMiS) proudly presents the BISMiS Live seminar series.

BISMiS Live has been a resounding success since its inception in March 2021. Our monthly sessions have engaged microbiology students, early career scientists, and seasoned experts alike. These interactive seminars explore diverse topics related to microbial ecology and systematics, featuring esteemed speakers from around the globe.

Upcoming Session Details:

Date: May 18th, 2024

Time: 0700 GMTFeatured Speakers:

Prof. William B Whitman (University of Georgia)

Dr. Luis M Rodriguez-R (University of Innsbruck)

Dr. Maria Chuvochina (University of Queensland)Session Title: “Proposed amendments to introduce Candidatus into the Code is not the best of both worlds”

The upcoming session is in response to the recent proposal regarding the integration of Candidatus into the International Code of Nomenclature of Prokaryotes that has ignited spirited discussions within our community.

Kindly refer to our website (https://www.bismis.net/bismislive.html) and share this announcement with your colleagues and peers. Your active participation enriches our discussions.

Kind regards,

Aaron,

Secretary of Bergey’s International Society for Microbial Systematics (BISMiS)

===

On behalf of

Wen-Jun Li, President

Stephanus Venter, President-elect

William Whitman, Treasurer

Kamlesh Jangid, BISMiS Live CoordinatorBergey's International Society for Microbial Systematics (BISMiS)

bismis.net

BISMiS - Bergey's International Society for Microbial Systematics

BISMiS - Bergey's International Society for Microbial Systematics

13 June – Wee Fei Aaron Teo

BISMiS Live brings to you another round of discussion on the proposal integrating Candidatus names into the ICNP (see https://doi.org/10.1099/ijsem.0.006188).

The session is scheduled for 22nd June 2024 at 0700 GMT. Please note the shift to 4th Saturday for this session. Speaker(s):

PD Dr. Markus Göker, Leibniz Institute DSMZ, Germany

Prof. Dr. Edward R.B. Moore, Culture Collection University of Gothenburg

Prof. Henrik Christensen, University of Copenhagen

Title: Further integrating Candidatus names into the ICNP is the best of both worlds

ZOOM Registration link: https://us02web.zoom.us/webinar/register/WN_r6gMRpGLR5KYMYu67VgOVw Please share the attached announcement of the 35th session with your peers and I look forward to seeing you all there. Thank you.

Kind regards,

13 June – Fanus Venter

Our commentary on the proposal has now been accepted for publication in Systematic and Applied Microbiology. Here is a copy of the manuscript.

14 June – Fanus Venter

After the ICSP Plenary I am still not sure how the list editors will deal with the Pallen et al 2022 (https://doi.org/10.1099/ijsem.0.005482) list of names. Including them in any Candidatus list to be published before the end of the year together with the approval of the rules in the BOBW proposal will have a major impact on taxonomic practices. We need clarity on this asap.

22 June – Fanus Venter

Based on feedback received it seems highly unlikely that the 65 000 names will be included in a Candidatus list before the end of the year. This raises the following questions should the BOBW proposal be accepted:

1) Does this mean that these names will not receive the status of pro-validly published (Rule 68 (2)) 2) What mechanism will will be put in place to deal with Candidatus names published before 2025 but that was not included in a Candidatus list? 3) Linked to the previous question How will the BOBW rules deal with the status of the 65 000 names if the decision not to include them in the *Candidatus* list, based only on work-load and resources to deal with it, is challenged? Would it be possible to still grant them the status of pro-validly published names as they were already published in 2022?

25 June – Markus Göker

As for (1), the current version of the BOBW proposal would require the 65,000 names to be listed in a Candidatus list before 2025 or require them to be proposed in an effective publication. As for (2), names from an effective publication before 2025 can be included in a Candidatus list published in 2025 or later. As for (3), neither the ICNP nor the current version of the BOBW proposal provide a mechanism for challenging inclusion in a Candidatus list before 2025. BOBW does provide mechanism for making them pro-validly published later on if they meet the requirement.

4 July – Fanus Venter

I still view the exclusion of the 65000 names as an inconsistent treatment of Candidatus names that have been published before the end of 2025. To be fair I would propose that you scrap rule 68 (2) and only consider Candidatus names published from 2025 onwards to be pro-validly published. You could recommend the re-use of the older Candidatus names but it will not be an enforceable requirement.

4 July – Markus Göker

Why should this be inconsistent? BOBW proposes to consistently treat each Candidatus name that occurred in a Candidatus List before 2025 as pro-validly published; those among them which do not adhere to the other criteria would be pro-illegitimate (and therefore could not be the pro-correct name of a taxon). Quite similar to the treatment of the Approved Lists in the ICNP. Quite a lot of work by Aharon Oren and others went into compiling these Candidatus Lists. Moreover, the Candidatus Lists would also be relevant after 2024 for pro-valid publication. For this reason, it appears to be more consistent and also easier to understand if Candidatus Lists had the same effect, whether until 2024 or after 2024. (edited)

22 June – Fanus Venter

It will also be of help to make an informed decision on the BOBW proposal if the following issues could be clarified. 1) If accepted as is will Candidatus names published after 1 Jan 2025 only be considered for pro-valid publication if the name, protologue and type material are listed as part of a publication and not in any supplementary material? 2) Will only names specifically indicated as Candidatus be considered under these rules or will names linked to effective publications (where the names could not be validated but an isolate was indicated as the nomenclature type) or linked to a genomes sequence also be considered for pro-validation?

22 June – Markus Göker

Thanks for these questions. Please note that, as a result of this debate, parts of the proposal can still be modified before putting it to the ballot. Apart from that, the answer to (1) is that our proposal does not change the definition of "effective publication"; it is up the ICSP to decide whether additional changes to this term should be made (I could think of one). The answer to (2) is no, because if the problem is just the missing term "Candidatus", this can be overruled based on Rule 68(3). Another clause of interest in this regard is Rule 68(4).

23 June – Alexandr Nemec

Dear all,

I would like to comment on the discussed paper by Pallen et al. 2022. I will not criticize the authors' (questionable in my opinion) rationale for the application of ≈45,000 artificial eubacterial names, but focus on the taxonomic data utilized by the authors, using the genus Acinetobacter as an example:

i. Sixty-two Candidatus species names were proposed for Acinetobacter with reliance on sequences and their classification and metadata available from the GTDB; 53 of them were derived from cultured strains and 9 from metagenomes [one MAG is not derived from Acinetobacter (according to 16S rRNA and gANIb) and at least three MAGs are contaminated).

ii. Of the 53 non-MAG sequences, 8 can be allocated to correctly named species, 1 to an only effectively named species, and 16 to tentative species with previously published provisional (non-binomial) names (e.g. https://onlinelibrary.wiley.com/doi/10.1002/9781118960608.gbm01203.pub2, https://apps.szu.cz/anemec/Classification.pdf or https://apps.szu.cz/anemec/Genomes.pdf). Moreover, some other sequences are genomically closely related to known species (the evidence of their status as separate species is missing).

iii. As many as 26 non-MAG sequences were derived from strains collected and studied by us or our collaborators; most were announced, analyzed, and interpreted in published articles. As explained in those articles, we did not propose binomial names for the sequenced strains because of the absence of taxonomically sufficient support for their circumscription at the species level. Yet, the sequences have been made publicly available to facilitate obtaining more comprehensive material needed for meaningful taxonomic conclusions. For the same reason, we designated most of them with provisional names.

iv. Thus, only a limited subset of the proposed Candidatus names label plausible sequences possibly representing Acinetobacter species unknown to the community including many sequences, which have been intentionally left formally unnamed based on insufficient taxonomic support for species circumscription, description and naming.

Although I understand (and accept) that my opinion on when and how to label novel taxa with formal binomial names (incl. Candidatus) fundamentally differs from that of Pallen et al., the above data cast doubt on their approach in scientific and ethical terms (similar problems for non-Acinetobacter sequences are expectable).

I anticipate the approval of the "The best of both worlds" proposal. If so, I do hope that the Candidatus names proposed by Pallen et al. will not be pro-validly published or it will not be done without proper taxonomic revision and appreciation of the work of the authors of the sequences.

Best regards,

Alexandr

Alexandr Nemec, Ph.D.

Professor of Medical Microbiology

Laboratory of Bacterial Genetics

National Institute of Public Health

Srobarova 48

100 00 Prague

Czech Republic

https://szu.cz/anemec

23 June – Mark Pallen

Good morning colleagues. I should like to thank those who have shown interest in my work and take this opportunity to take the discussions forward.

However, let me start by clarifying my own position. I do not support “the best of both worlds” proposal. I was invited to contribute early on to this initiative, but quickly withdrew my support because I saw problems in principle and in practice with the proposal.

On the practical level, I find the use of the neologism prefix “pro“ as aesthetically unpleasing and confusing.

But more fundamentally, if one says that Candidatus species and high-level names must be re-used when a representative strain is cultured, this is effectively saying that they have the same standing as validly published species names, which has already been rejected within the ICNP.Furthermore, it fails to resolve the problem of what happens when a cultured representative is grown from Candidatus phylum named after a Candidatus genus, but where the cultured strain belongs to a different genus. Does the phylum name have to change or not?

And if one simply says that Candidatus names should be re-used unless there are good reasons not to, how does this change the current situation, where the presumption is already “aim for stability of names”?

23 June – Markus Göker

As for "On the practical level, I find the use of the neologism prefix “pro“ as aesthetically unpleasing and confusing.", this seems subjective. If another prefix is considered preferable, it can be suggested. Furthermore, the idea of using prefixes should be clear: they clarify the analogy to already established status values used by the ICNP. This also confirms that the proposal is not really complicated: instead of 3 status values of interest, there would be 6, and the 3 new ones would be formed in analogy to the 3 known ones. This is so simple that it can be explained in a single, simple figure (Figure 1 in our manuscript).

23 June – Markus Göker

As for "But more fundamentally, if one says that *Candidatus* species and high-level names *must* be re-used when a representative strain is cultured, this is effectively saying that they have the same standing as validly published species names, which has already been rejected within the ICNP", I'm sorry to say that this is simply factually incorrect. It is clear from our proposal that pro-validly published and validly published are different things. The proposal says so explicitly, and it spells out the different consequences of each nomenclatural status. This is clear, for example, when a pro-validly published and pro-legitimate name is considered to be synonymous with a validly published and legitimate name. It cannot be emphasised enough that our proposal is backward compatible and does not conflict with any previous ICSP decision. The 2020 ICSP decision refused to give "validly published" status to names based on genome sequences as the nomenclatural type. The BOBW doesn't do that either. And this is not just a difference in labelling. The BOBW proposal explains in detail the differences in meaning and consequences of each nomenclatural status. Another misrepresentation of our proposal is that it "says that Candidatus species and high-level names must be re-used" -- instead the proposal says that pro-validly published and pro-legitimate Candidatus names must be reused under certain conditions.

23 June – Markus Göker

As for "Furthermore, it fails to resolve the problem of what happens when a cultured representative is grown from Candidatus phylum named after a Candidatus genus, but where the cultured strain belongs to a different genus. Does the phylum name have to change or not?", this is not only explained in the BOBW paper but was also treated in detail in the talks given in the ICSP plenary meeting and also in BISMiS Live. This refers solely to proposed Rule 72(4), which already clarifies the alleged problem, and if the current version of proposed Rule 72(4) is deemed unsatisfactory, the ICSP can vote separately on 72(4). The authors can also provide alternative versions for it.

23 June – Markus Göker

As for "And if one simply says that Candidatus names should be re-used unless there are good reasons not to, how does this change the current situation, where the presumption is already “aim for stability of names”?", the ICNP does not say "Candidatus names should be re-used unless there are good reasons not to". Acceptance of BOBW would clarify that. Importantly, "aim for stability of names” is a (part of a) Principle, not a Rule. The actual implementation of the Principles is found in the Rules. The Principles alone don't tell you what to do. Moreover, reuse is only one aspect of many. One must not overlook the other issues clarified by BOBW, such as the avoidance of homonyms and the regulation of which Candidatus name to apply in the case of assumed synonymy.

23 June – Mark Pallen

I have reviewed the status of the Candidatus approach here: https://www.microbiologyresearch.org/content/journal/ijsem/10.1099/ijsem.0.005000

A key point from that paper is:

"An alternative view is to see the absence of formal standing for Candidatus names not as a 'bug' but as a 'feature', as this means that they could be applied as provisional names to uncharacterized taxa, as an alternative to cumbersome alphanumeric labels, while leaving open the option that they

could subsequently be changed after further characterization of taxa or changes in opinion."If re-use of Candidatus names is mandated, this flexibility is lost, which I think would be a shame.

23 June – Markus Göker

First of all, not all Candidatus names would need to be reused under BOBW. The proposal defines which ones would have to be reused and under which conditions. The proposal also clarifies which Candidatus name would need to be applied in case of synonymy, which is an important aspect irrespective of the reuse question. It also addresses the issue of homonymy. Moreover, under BOBW names could still be changed "after further characterization or changes in opinion". For instance, if one opined that a genus should be split or some genera should be merged, this would imply proposing name changes (new combinations). However, if no such thing is happening, the aim for stability of names (which must always be read in conjunction with the other aims of Principle 1) would call for reusing a Candidatus name upon valid publication, if it is believed to represent the same taxon.

24 June – Mark Pallen

Moving on to discussion of the 65,000 arbitrary names we have released in a peer-reviewed publication in the ICNP house journal IJSEM (https://www.microbiologyresearch.org/content/journal/ijsem/10.1099/ijsem.0.005482), in that paper we clearly state:"The fact that Candidatus names have no standing in nomenclature is often seen as a deficiency of the current system of bacterial nomenclature. However, the provisional status of the names proposed here can be seen as helpful, in that if, in the future, those working on a given taxon wish to propose a new name, they are perfectly entitled to do so within the current system—albeit with the proviso that the opening principle of the ICNP is to 'aim for stability in names'. Aside from such changes, the vast majority of the Candidatus names proposed here are likely to remain highly stable—given that only 0.26% of genomes are on average assigned to a different species cluster from one release of GTDB to the next—and so can now be safely adopted by databases and used in the scientific literature."There was never any intention to force people to use these names if they don’t want to. My goal in this work was simply to provide user-friendly alternatives to the non-human-friendly alphanumeric labels applied to unnamed taxa in GTDB. (edited)

24 June – Mark Pallen

In my experience, this has already proven useful, for example in this table from our paper on the oesophageal microbiome: https://www.ncbi.nlm.nih.gov/pmc/articles/PMC10323806/table/T1/?report=objectonlyWhere IMHO Ca. Alloprevotella rovamia is a lot easier to remember than Alloprevotella sp000318095.Similarly, I just finishing supervising an undergraduate project looking at the phylum labelled UBA9089 in GTDB, which is a lot easier to remember and discuss if the phylum is instead called Ca. Omexiota. In fact, I just had to look up UBA9089, whereas Omexiota was already easily available within my working memory.

PubMed Central (PMC)PubMed Central (PMC)

An initial genomic blueprint of the healthy human oesophageal microbiome

The oesophageal microbiome is thought to contribute to the pathogenesis of oesophageal cancer. However, investigations using culture and molecular barcodes have provided only a low-resolution view of this important microbial community. We therefore explored ... (174 kB)

https://www.ncbi.nlm.nih.gov/pmc/articles/PMC10323806/table/T1/?report=objectonly

11:57

My motivation in applying well-formed user-friendly names to unnamed taxa stems from the belief that having names for taxa that have already been defined and circumscribed creates a positive feedback loop that encourages and facilitates further study at the cutting edge of discovery. The fact that over 80% of species defined by GTDB lack well formed names is proof that current approaches to nomenclature are failing to keep up with the pace of discovery: https://gtdb.ecogenomic.org/stats/r220

But perhaps more concerning is that the majority of bacterial phyla lack well-formed names: https://gtdb.ecogenomic.org/stats/r220#nomenclatural-types-per-rankAnd it seems to me clear that phylum names like Ca. Dobariota and Ca. Soduniota are less confusing and easier to remember than p__UBA6262 and p__UBA6266.

24 June – Mark Pallen

Now let me move on to the specific points raised by Fanus Venter and Alexandr Nemec.In applying arbitrary names to tax already defined by GTDB, I am working with the taxonomic opinion of the GTDB team as to what counts as a taxon. If others disagree with the GTDB definitions, that is not a matter of nomenclature but taxonomy and as the Code states nothing in the Code restricts taxonomic freedom of thought.Venter and Nemec both touch on the fact that they think that only those who culture a type strain have the right to name the species or other taxa associated with it. But the ICNP specifies no such right. And as we note in the paper:

"The scale of our efforts here, together with the fact that we are naming taxa that have already been delineated and classified by others, begs the question 'Who has the right to create and assign taxonomic names?' Although the ICNP says nothing on this issue, traditionally the task of naming newly cultured species has fallen to those who isolate and discover the species and deposit type material in culture collections. As this often requires a substantial effort, the act of naming can be seen as a reward for the 'sweat of one's brow'. However, it remains unclear whether similar principles can be applied to the naming of uncultured species defined only by sequence analysis, when who can say who should be rewarded with a stake in the process: those who collected samples, those who sequenced them, those who binned reads into metagenome-assembled genomes or those who performed the sophisticated phylogenetic analyses delineating and classifying uncultured taxa? In any case, so far, none of these parties has shown interest in—or developed competing methodologies for—creating new names at scale."Christensen does raise an interesting point in highlighting that we are applying Candidatus names to cultured isolates. This is discussed in both papers:"According to the strictest interpretation, Candidatus names can be assigned only to uncultured taxa and, here, we have made the assumption that placeholder names in GTDB are generally associated with uncultured taxa. However, as Pallen [14] has argued elsewhere, even if some renamed taxa do have cultured representatives, we can safely fall back upon the broader definition of Candidatus as a category 'used for describing prokaryotic entities. for which characteristics required for description according to the Code are lacking'."

"However, this brings to mind a deeper philosophical question: should the status of names depend on cultivability? This can be seen to conflict with the opening principle of the ICNP: 'Nothing in this Code may be construed to restrict the freedom of taxonomic thought or action' and with General Consideration 5: 'This Code of Nomenclature of Prokaryotes applies to all Prokaryotes' (my

italics). In addition, the requirement to use the term Candidatus has not been applied retrospectively, so it does not apply to the names of uncultured species published in the Approved Lists of Bacterial Names or to the thirty or more validly published names of uncultured organisms approved between 1980 and 2001 [4, 49–67]. These glaring inconsistencies sit uneasily within what is supposed to be a precise rule-governed system of nomenclature.

However, there is also a serious operational issue at stake. According to the strictest interpretation, assigning a Candidatus name to a taxon depends on proving a negative—being confident that the taxon has never been cultured by anyone anywhere in the world. To take a lively example that applies at the time of writing, let's say we wished to assign a new Candidatus name to the genus given the designation CAG-485 by GTDB (https://gtdb.ecogenomic.org/searches?s=gt&q=CAG-485). Almost all of the more than 100 genomes classified within this genus represent metagenome-associated genomes, so it might appear safe to propose a new Candidatus name. However, only after exhaustively working through the metadata associated with all of the BioSamples associated with these genome sequences in the NCBI databases does it become clear that at least one of these (NCBI BioSample SAMN10878315) is in fact derived from a cultured isolate, so that some might argue the status Candidatus cannot be applied. A similarly exhausting process awaits anyone attempting to prove that well-established Candidatus taxa do not yet contain cultured relatives. In all such cases, it is probably best if we fall back upon the looser definition of Candidatus, 'used for describing prokaryotic entities … for which characteristics required for description according to the Code are lacking' and deny Candidatus status only when someone provides proof of culture in a peer-reviewed publication.

Scrutiny of genome sequences assigned to CAG-485 reveals an additional problem. Several of these originate from a study of the mouse gut microbiota conducted in Germany and are tagged as derived from cultured isolates [68]. However, on reading the paper it becomes clear that these represent genomes from 'strains that could be isolated but failed being maintained in culture'. Should taxa based on such criteria be allowed Candidatus status? If so—as complying with the requirements for effective and valid publication of names for cultured taxa is far more time-consuming than publishing Candidatus names—those interesting in cataloguing microbial diversity may well favour approaches that avoid stable culture altogether and simply assign Candidatus (or even SeqCode) names to newfound organisms."And finally, I have tried this morning to investigate the claims made by Nemec but have failed to do so, as I cannot unequivocally identify the taxa and genomes that he claims have already been provisionally named. There are several reasons for this. PDFs are hard to parse into tabulated data. NCBI uses different IDs for the same sequence, so simply searching for the genome IDs in GTDB fails to work. But in any case, the ICNP does not define any right to say that a species cannot be given a name by anyone other than the person depositing a genome sequence.

24 June – Mark Pallen

Please explain in pure and simple terms how this takes us beyond the current status quo in use of Candidatus, apart from clarifying that Candidatus names should be published in the same way as conventional names? And also if the names have to re-used on culture, how can we justify forcing the hand of those who have put the effort into making the culture work? And what happens to names of higher level Candidatus taxa when a genus other than the type genus is cultured?

I see the section on Candidatus Dedyshiibacter, but this does not resolve the stability in names problem if a genus with one name is renamed with a genus name already in use for another genus. This is a source of confusion not clarity.

24 June - Markus Göker

I am beginning to suspect that not only is the BOBW paper TLDR for you, but so are my responses to your comments here on Slack :wink: To reiterate: BOBW clarifies the avoidance of homonyms between pro-validly published and pro-legitimate Candidatus names on the one hand, and validly published and legitimate names on the other, clarifies the reuse of pro-validly published and pro-legitimate Candidatus names upon valid publication of a name for the same taxon, and clarifies which Candidatus name to apply when multiple Candidatus names are considered synonymous. Obviously, none of this is currently regulated in the ICNP. BOBW adds the missing provisions for Candidatus names. As for "if the names have to re-used on culture, how can we justify forcing the hand of those who have put the effort into making the culture work?" this is not only easily justifiable, but one has to keep in mind that the BOBW proposal results in an incentive to propose Candidatus names, while maintaining an incentive to validly publish names (and thus cultivate and deposit strains). BOBW distinguishes between 6 instead of 3 status values in order to obtain a more informative code, which is also linked to maintaining an appropriate motivation in nomenclature. As for "And what happens to names of higher level Candidatus taxa when a genus other than the type genus is cultured?", this has been explained three times now (four if you include the BOBW paper itself), and I'd suggest returning to this topic when a question is asked that has not yet been answered. (edited)

24 June - Markus Göker

As for "I see the section on *Candidatus* Dedyshiibacter, but this does not resolve the stability in names problem if a genus with one name is renamed with a genus name already in use for another genus. This is a source of confusion not clarity", this issue has been addressed three times now, once in my talk at the ICSP plenary, once in my BISMiS Live talk, and once here on Slack. To reiterate: This only concerns the proposed Rule 72(4), which is not part of the core of the proposal. The ICSP can vote on 72(4) separately and/or the authors can provide another version of it before putting it to a vote. (edited)

5 July - Markus Göker

I failed to add that there appears to be a logical contradiction between different but connected comments on reuse or non-reuse of Candidatus names. On the one hand we find "And also if the names have to re-used on culture, how can we justify forcing the hand of those who have put the effort into making the culture work?", which means authors should rather not have to reuse Candidatus names (because we can not really "justify forcing [their] hand"), although this also means in practice that authors are allowed to create synonyms or homonyms. However, on the other hand we find "I see … this does not resolve the stability in names problem if a genus with one name is renamed with a genus name already in use for another genus. This is a source of confusion not clarity", which means authors should rather have to reuse Candidatus names upon cultivation (because if otherwise this would be a "source of confusion", probably because of the possible creation of synonyms or homonyms). The first of the two comments sees "stability in names" as rather unimportant,

but in the second comment "stability in names" is rather important again. Doesn’t look consistent to me.

I think BOBW is actually quite clear about is criteria for reuse, with the only exception of interest being proposed Rule 72(4), which is already phrased quite carefully. The pros (increased incentive to cultivate and deposit the first strain belonging to a higher taxon) and cons (potential of renaming) should be obvious. As said several times, this Rule can be voted on separately, and the BOBW authors may create alternative versions based on the comments received.

24 June – Mark Pallen

Actually I neglected to say that another key reason why I did not want to be associated with the BOBW manuscript was the highly polarised and polemic use of language with regard to the SeqCode, which I do not find agreeable or helpful and added to the TLDR nature of the manuscript.

24 June – Markus Göker

You did not attend the last ICSP plenary meeting, or at least the first part of it. At that meeting an introductory talk was given for new ICSP members, in which it was reiterated what the functions of the ICSP are, with reference to the Statutes. One of the two functions is to ensure the proper application of the ICNP. Published misunderstandings of the ICNP have a negative impact on the proper application of the ICNP and therefore need to be addressed by the ICSP. Of course, there are not sufficient resources to address all misconceptions, but if the ICSP can address the most prominent ones, it should do so. It's a statutory obligation. The same goes for prominent actions that are in direct contravention of the ICNP. Such actions should also be highlighted. Perhaps the statutes are also TLDR for some ICSP members, which may explain the confusion. However, the ICSP-EB has recently been more active in addressing misunderstandings of the ICNP and the ICSP, both within and outside the ICSP itself. (edited)

25 June – Barny Whitman

This alternative proposal was developed by Maria Chuvochina, Phil Hugenholtz, Kostas Konstantinidis, Luis-Miguel Rodríguez Rojas, Iain Sutcliffe, Fanus Venter, and Barny Whitman

The discussions on Bismis Live on May 18 and June 22 were very informative. For the reasons presented in these discussions and Whitman and Venter (2024), we urge the ICSP to reject the Section 10 proposals. Even if you don’t like the SeqCode, Section 10 offers only a partial solution and will be a challenge to administer.

An alternative is to use the SeqCode as a resource for the ICSP/ICNP. If the ICNP recognized SeqCode names as pro-validly published, the SeqCode rules and Registry could provide a resource for establishing priority among Candidatus names. For instance, the SeqCode has a system in place for establishing data standards to ensure that ambiguous and useless names are not created. A group within the SeqCode community is also currently examining existing Candidatus names to determine which ones meet the data requirements for registration. On its side, the SeqCode could be amended to recognize paratypes, which would be isolated strains representative of the taxon. As paratypes become available, SeqCode names would become validly published under the ICNP. Potential problems with this arrangement could probably be solved by discussions between representatives of the ICSP and the SeqCode committee.

This really would be the best of both worlds.

3 July – Markus Göker

As for "This alternative proposal was developed", frankly I see no developed alternative proposal here at all.

3 July – Markus Göker

As for “The discussions on Bismis Live on May 18 and June 22 were very informative. For the reasons presented in these discussions and Whitman and Venter (2024), we urge the ICSP to reject the Section 10 proposals”, our presentation at BISMiS Live on 22 June, we reiterated the problems caused by SeqCode and also provided counter-arguments to the criticisms of BOBW. These criticisms of BOBW are either factually inaccurate, exaggerated, or things that could easily be fixed in BOBW if they were considered important. The same applies to additional criticisms raised in Whitman and Venter (2024). The authors of the BOBW will respond to this paper in due course.

3 July – Markus Göker

As for “Even if you don’t like the SeqCode, Section 10 offers only a partial solution and will be a challenge to administer.”, no evidence was provided that Section 10 offers only a partial solution. It will not be a challenge to administer either. Most of the real work has to be done by the IJSEM List Editors, the Judicial Commission, and databases such as LPSN. You may notice that a good deal of the people involved in this work are co-authors of the BOBW paper. For example, all three commissioners of the JC are co-authors. So, complexity is not really an issue. As for the "community", the BOBW paper describes in detail how little additional effort would be needed for this “community” if BOBW got implemented.

3 July – Markus Göker

As for “An alternative is to use the SeqCode as a resource for the ICSP/ICNP. If the ICNP recognized SeqCode names as pro-validly published, the SeqCode rules and Registry could provide a resource for establishing priority among Candidatus names”, this statement is in obvious logical conflict with the previous statements. Only the BOBW defines the status of "pro-validly published"; therefore, if the BOBW is not ratified, the ICNP cannot recognize "SeqCode names as pro-validly published". As we made clear in our BISMiS Live presentation and also in the ICSP plenary, if BOBW is implemented, SeqCode "validly published" names can be recognized as pro-validly published under ICNP. Moreover, BOBW itself provides the mechanism to establish pro-priority among Candidatus names. And the ICSP has already a mechanism in place to register Candidatus names.

3 July – Markus Göker

As for “On its side, the SeqCode could be amended to recognize paratypes, which would be isolated strains representative of the taxon. As paratypes become available, SeqCode names would become validly published under the ICNP.” this seems also irrelevant, as names “validly published” under the SeqCode can already become validly published under the ICNP, provided the requirements of Rule 27 and the Rules it cites are fulfilled; the name then may or may not refer to the same taxon.

3 July – Matthew Stott

Good evening everyone.

I wish to add to the discussion on the BOBW proposal, and state at the outset, I will not be supporting the proposal. My reasoning is two-fold: I agree with the commentary by Barney above, and with the comments in the recent Whitman and Venter (2024) manuscript. But secondly, I consider that the multi-tiered pro-validated system for DNA as proposed by BOBW as overly complex, confusing and is unlikely to attract the general microbiology researcher community to the system. This complexity stems from trying to develop a system that places an unnecessary hierarchy (priority) of isolates over DNA as type material.

I say ‘unnecessary’ here not negate the arguments made in the BOBW proposal, but to remind us that the goal for the ICSP is to create and maintain mechanisms in the Code that are accessible by the broad microbiology researcher community (for whom we serve) and to facilitate that community to more easily communicate the mind-blowing diversity and importance of the microbial world that we all love and are passionate about. I don't believe that installing this hierarchy promotes this communication, and is more likely to serve as a barrier.

Some background:

As disclosure, I was one of the attendees of the workshop for the Roadmap manuscript [Murray et al., 2020] that proposed the SeqCode as one of the possible paths to addressing the issues around the taxa unable to be deposited in international culture collections (I highlight the ‘one of the possible’ paths here and will come back to this below). I note here that I am not associated with any of the downstream publications on the SeqCode, nor am I associated with the SeqCode Committee. I am passionate about cultivation and describing microorganisms (both cultivated and uncultivated). I have published microbial names via the ICNP and the SeqCode (and have added to the general chaos by also proposing names via neither code) and I research in microbial ecology, systematics, and applied microbiology spaces.

Coming back to the original Roadmap manuscript and proposal. I can categorically state that in the discussions at the Roadmap manuscript workshop, that the formation of a separate code (in the end, the SeqCode) was the backup option and the preferred option was always to try and convince the ICSP to amend the Code to use DNA as type material with equivalent priority status. As you all know, that proposal was rejected in 2020. I can also say that at the workshop, we also considered that if an alternative code was created, then the end goal should always be to unify back to a single code.So, my take on the BOBW is that it is a positive step in the right direction towards that unification, but I think the issues described by Whitman and Venter (2024) make the proposed emendments to Section 10 of the Code problematic. But equally importantly, I think it won’t convince the general microbiology researcher community that it is a better (easier, more accessible, more understandable) option than the SeqCode or the wildwest alternative.

I see the easiest and best solution as conferring the same priority status to high quality genomic DNA as a deposited strain. I disagree that by doing this, we discourage microbiologists from attempting to isolate strains. Despite the presence of the SeqCode or general wildwest of last decade, I highlight the amazing work to isolate representatives of the Lokiarchaeota and the umpteen publications on new techniques to grow hard-to-cultivate strains.

I will not waste your time by repitching many of the arguments made by others at BiSMiS, in this Slack Forum, in the various publications; they are more eloquent than I could ever muster. (Although I do highlight that the BOBW proposal seems to lack strong criteria of what high quality DNA type material is (I point people to the definitions by Hedlund et al., 2022, Table 3 as a good example of what this should look like)).So, while I am voting to reject the BOBW proposal, I am doing so with a positive outlook that the proposals and discussions here are moving in the right direction towards a usable system. My hope is that we are not far away from a mechanism that is agreeable to all and that a really positive step would be for the Exec Committees of the ICSP and SeqCode to sit down together and sort out a solution.

3 July – Markus Göker

Thanks for the long comment. However, like many some other comments it has the deficiency that it does not refer to the BOBW paper itself but only to comments made by BOBW-hostile third parties. For instance, it seems that some supporters of the SeqCode are not seeing BOBW as the compromise it actually is but stubbornly insist on the literal implementation of the SeqCode. Given that the SeqCode supporters not normally respond to the many criticisms raised against the SeqCode, this does not make a good impression to me. Nor do your comments address any of those issues.

3 July – Markus Göker

One of statements against BOBW made here is this issue of alleged complexity. As for, "I consider that the multi-tiered pro-validated system for DNA as proposed by BOBW as overly complex, confusing and is unlikely to attract the general microbiology researcher community to the system," we need to remember that BOBW mainly adds three terms (pro-validly published, pro-legitimate and pro-correct) to three other, established terms (validly published, legitimate pro-correct), and that the three new terms are formed in analogy to the three previous ones. This does not really add much complexity, let alone complexity that a scientist cannot bear. Most of the real work has to be done by the IJSEM List Editors, the Judicial Commission, and databases such as LPSN. You may notice that a good deal of the people involved in this work are co-authors of the BOBW paper. For example, all three commissioners of the JC are co-authors. So, complexity is not really an issue. As for the "community", the BOBW paper describes in detail how little additional effort would be needed for this “community” if BOBW got implemented.

3 July – Markus Göker

As for "I don't believe that installing this hierarchy promotes this communication, and is more likely to serve as a barrier.", we need to remember that the ICNP already distinguishes between Candidatus names and validly published names, so BOBW does not even introduce this hierarchy. In contrast, BOBW gives higher value to certain Candidatus names as before while at the same time not changing the Candidatus concept itself (as has erroneuosly been claimed sometimes).

3 July – Markus Göker

As for the term "priority", this is a term defined by the ICNP, and the way you use it here is different from the way the ICNP defines it. This may only add to the confusion.

3 July – Markus Göker

As for “I will not waste your time by repitching many of the arguments made by others at BiSMiS, in this Slack Forum, in the various publications; they are more eloquent than I could ever muster.”, I wonder why you are not referring to the counterarguments provided by the BOBW authors (including in their BISMiS Live session) and the vast majority of the arguments against the SeqCode. As for “the BOBW proposal seems to lack strong criteria of what high quality DNA type material is”, this is one of the points that can easily be added to BOBW if it is of interest, as highlighted by the BOBW authors at several occasions.

3 July – Markus Göker

As for "I see the easiest and best solution as conferring the same priority status to high quality genomic DNA as a deposited strain. I disagree that by doing this, we discourage microbiologists from attempting to isolate strains.", you make a mistake that has been made by supporters of SeqCode on BISMiS Live: you fail to see that it is not just about isolating, but about isolating and depositing. Only then will the strains be available for future generations and other researchers will be able to replicate the author's research. Furthermore, you miss the point that the incentive to isolate and deposit is not removed in general, but from the nomenclature. As the ECCO commented in 2020 on Whitman's proposal to accept DNA sequences as nomenclatural types of species or subspecies with a validly published name: "If the proposal is accepted, it will no longer be strictly necessary to deposit any material in public collections, be it type-strains, DNA-extracts or environmental samples. This of course does not mean that we think that efforts to isolate and study new species in culture would be completely discarded nor that all scientists who have regularly been depositing strains will suddenly stop to do so, but overall the motivation to do all that is needed for depositing type-strains in a public collection will decrease. The proposal allows for later replacement of a type sequence by a type strain when the latter becomes available, but in our experience, we believe that in practice this will be forgotten or, worse, simply ignored. This should be a major concern to all stakeholders, especially in the light of climate change and the accelerated loss of biodiversity."

3 July – Markus Göker

As for “I am passionate about cultivation and describing microorganisms (both cultivated and uncultivated). I have published microbial names via the ICNP and the SeqCode (and have added to the general chaos by also proposing names via neither code) and I research in microbial ecology, systematics, and applied microbiology spaces”, it must be remembered that BOBW creates an incentive to suggest names for uncultivated prokaryotes and also maintains an incentive to cultivate, deposit and name those cultivated prokaryotes. For this reason, BOBW is the fair solution. Giving equal status to names based only on a MAG and names based on a deposited culture is not.

3 July – Markus Göker

As for “So, my take on the BOBW is that it is a positive step in the right direction towards that unification, but I think the issues described by Whitman and Venter (2024) make the proposed emendments to Section 10 of the Code problematic” one must not overlook that already the BISMiS talks by us on June 22 refuted the counterarguments made in the

previous BISMiS talk. The BOBW authors will respond to additional issues raised by Whitman and Venter (2024) in due course. As for “But equally importantly, I think it won’t convince the general microbiology researcher community that it is a better (easier, more accessible, more understandable) option than the SeqCode or the wildwest alternative”, this lacks evidence. More importantly, it is affected by what could rightfully be called the “easiness ideology” or “simplicity ideology”. Why considering this ideology? Just one example: The Ancient Greeks distinguished between four elements: air, earth, fire and water. Now we distinguish between more than 100 chemical elements. This is, of course, not the “easier, more accessible, more understandable” option than the four elements. And yet we choose it. Why? Because it is more informative. Similarly, BOBW results in a more informative code any attempt to give names based on MAGs and names based on an isolated and deposited strain the same status in nomenclature.

4 July – Fanus Venter

As for "For instance, ask yourself whether the Whitman & Venter paper mentions any of the criticisms that have been raised against the SeqCode in the BOBW paper .." Good question to ask and the direct answer is that the focus should be on the Section 10 proposal and its implications and not on differences in opinion on what should be considered to be suitable material to serve as a nomenclature type. This question has previously been debated and all we can agree on is that we have a difference in opinion. No use for further lengthy papers or answers most microbiologists would consider to be TLDR.

4 July – Markus Göker

I was under the impression that earlier in this thread the TLDR attitude was occasionally worn with pride. But is it scientific? I suppose if it's your professional duty to judge something, you'd better study it thoroughly... But there's another problem here, because the TLDR "argument" was used by precisely those posters who at the same time suggested that Pallen et al.'s 65,000 Candidatus names be included in a Candidatus list. But isn't that list of 65,000 Candidatus names a prime example of a text to which the TLDR "criterion" would be applied? It seems that some people like the TLDR "criterion" more when it saves them some work, but not so much when it saves others some work... (edited)

4 July – Fanus Venter

Please explain: "So, neither is it necessary to include the 65,000 names in a Candidatus List right now". I am wrong in thinking that by not including them now (for no good reason apart from the fact that it is a lot of work and we don't like them) they will not be considered in future for pro-validation because if the Section 10 rules are implemented they would not be considered to be effectively published as these names formed part of the supplementary material?

4 July – Markus Göker

Yes, exactly. This, of course, also constitutes a good reason for not trying to include these names in a Candidatus List during 2024. But the same names could be proposed later in a document that meets the criteria for effective publication.

4 July – Matthew Stott

Hello Marcus, hello all

You make some sound arguments, many of which I agree with Marcus.I also don’t think anyone will disagree that the goal is to have a single system for nomenclature that is used and is accessible to a majority of researchers.

In order to achieve this, the BOBW needs to be up taken by a majority of the community, and thus has to offer something (that they believe) is superior than the options currently available.

So the question is, Does it?Because the risk is that if it doesn’t, then at best, we are no better off than we are now and at worst, it gets more chaotic.

If I take a global view, the idea that the proposal incentivises cultivation doesn’t sit well with me nor do I think it actually provides any incentive or that there is evidence that it does incentivise (and yes, there is a burden of proof to demonstrate it does, or conversely, demonstrate that cultivation and deposition because of a lack of incentive are decreasing if this claim continues to be cited).The hypothesis of incentivisation in this situation relies on researcher’s capability and means to do the said cultivation, isolation and characterisations. There are plenty of reasons why this can't necessarily be undertaken – legislative, indigenous rights and data sovereignty, access to capital equipment, trained staff and students, money, and so on… these are all barriers – what makes using DNA is that it democratises / increases the ability of microbiologists globally to participate. – all you need is an ability to extract DNA and online access. This is fantastic - the more people are engaged, the more microbiology is advanced!So given the above, what would the researcher community (some with potentially limited means) choose? The option where your nomenclature choice can be superseded because a priority ruling (i.e. the issues around rule 72(4)), or, having it recognised by the SeqCode as having equivalent status? The reality I suspect is most will go for the later or opt for neither and go with the wildwest status quo. If this is the case, then are we, as a community, in a better position?The ICSP has an obligation to provide the best system that provides utility to a majority of whom we represent.I just can’t see the BOBW doing this (...we are close, but not quite there).For this reason, I restate I do not think it should be accepted in its current form (but am positive that we're no far away).Post open comment period, it would good to see how the authors intend on addressing issues around rule 72(4) and their proposal around genome quality minimal standards.

Finally, if the proposal is accepted, then perhaps we as a committee should commit to measuring the impact of these changes so we some hard data demonstrating uptake? (edited)

4 July – Markus Göker

I think one of the main caveats here is not to decontextualize this debate. Much of the support for the SeqCode appears to be characterized by what is not mentioned rather than by what is mentioned. (For instance, ask yourself whether the Whitman & Venter paper mentions any of the criticisms that have been raised against the SeqCode in the BOBW paper ...) When you are asking “In order to achieve this, the BOBW needs to be up taken by a majority of the community, and thus has to offer something (that they believe) is superior than the options currently available. So the question is, Does it?” then I wonder why you don’t also ask the question about the SeqCode – a code that outright contravenes another code by construction and even attempts to redefine the scope of that other code! Has something like this ever happened in nomenclature before? It was clear from the outset that there would be people who wouldn’t accept the SeqCode. If “the goal is to have a single system for nomenclature that is used and is accessible to a majority of researchers”, why

even propose a second code in conflict with the existing one? I suggest you direct those questions towards the SeqCode in the first place. (Much like: "commit to measuring the impact of these changes so we [sic] some hard data demonstrating uptake".)

And: no, it doesn't help emphasizing that publishing another code was not your preferred choice. Publishing the SeqCode was not the only option once the ICNP had rejected the 2020 Whitman proposal. One could as well have published a code for sequences (not: organisms) that uses names or identifiers that can easily be distinguished from ICNP names, or one could have gone for a BOBW-like solution right away. (These issues, like many others, were detailed in the BOBW paper -- I'm not sure whether you've read it) But none of this was proposed. It seems it was just all-or-nothing, black or white. I am pretty sure many people already recognize BOBW instead as the feasible compromise it actually is.

But, apart from that, you may misconstrue what the "community" is. Most people involved are passive users of taxon names, they don't propose names (and you can't blame them for that). You don't know the majority of these people and you can't speak for them. Those who run the established institutions of nomenclature, particularly the IJSEM and some databases, do a good deal of the work for them. Quite a few names already have the Candidatus prefix, and under BOBW they will retain it. This does not negatively affect anyone. In contrast, it provides more information in the name than a system that blurs the distinction between names based on a deposited culture and names based on, e.g., only a MAG. Many people have already used the Candidatus concept. It is obviously "used and accessible to a majority of researchers". BOBW builds on this in a backwards compatible way by carefully adding further useful regulations.

4 July – Markus Göker

As for "Because the risk is that if it doesn't, then at best, we are no better off than we are now and at worst, it gets more chaotic", I have given an outlook on the future in the ICSP plenary and on BISMiS Live. I'm not sure whether you have considered that (as you consistently neither refer to the BOBW paper in any way nor to the other information provided by the BOBW authors). Four options were differentiated, and it was indicated that even if the SeqCode persisted, the situation would be improved by implementing BOBW. It was also explained why. But I appreciate that you admit that the current situation is chaotic (apparently because of the SeqCode).

4 July – Markus Göker

As for "The hypothesis of incentivisation in this situation relies on researcher's capability and means to do the said cultivation, isolation and characterisations. There are plenty of reasons why this can't necessarily be undertaken – legislative, indigenous rights and data sovereignty, access to capital equipment, trained staff and students, money, and so on… these are all barriers", this means you are admitting that cultivation and deposit is often difficult. Yet cultivation and deposit are important to let other researchers replicate your research and make cultures available for future generations. In order to actually "take a global view", one needs to think in longer terms, not just "I want to propose a name right now as quickly as possible and call it validly published", which is enourmously shortsighted. If something is difficult to do but useful for others and in the long term, one needs an

incentive to get people to do it. So, what you are saying here clearly demonstrates that an incentive to cultivate and deposit is needed in nomenclature.

Is is an established fact that the incentive in nomenclature to cultivate and deposit is that these are requirements for getting a name validly published under the ICNP. Obviously, the SeqCode removes this incentive from nomenclature because under the SeqCode you can also call a name “validly published” if it is just based on a genome sequence. This may either be a MAG or a genome sequence obtained from a culture; the SeqCode does not differentiate. The only way to retain this incentive in nomenclature is that the ICSP does not accept names “validly published” under the SeqCode as validly published under the ICNP. Another established fact is that BOBW does not change the criteria for valid publication. (Just read our proposal instead of relying on claims of third parties.) So, if getting a name validly published is the incentive in nomenclature to cultivate and deposit, and if BOBW does not change the criteria for valid publication, BOBW obviously retains the incentive to cultivate and deposit. No further proof is needed. However, who exactly has the burden of proof?

As for the burden of proof, it is again necessary not to decontextualize this. Given the well known background information, those who want to remove the incentive from nomenclature have the burden of proof. The SeqCode supporters must demonstrate that the SeqCode does not have this effect. We are talking here about potential long-term negative effects. So, the idea “there is a burden of proof to ... demonstrate that cultivation and deposition because of a lack of incentive are decreasing if this claim continues to be cited” seems quite reckless. Apart from that, we have already examples. For instance, there is a SAM case in which a name was “validly published” under the SeqCode just because the authors didn’t have the 2nd deposit in a culture collection. The SeqCode is not the “code for the uncultivated”, although it is often advertised as such.

Some people support the SeqCode because legal restrictions in their countries hinder them making their cultures accessible to other researchers. The SeqCode does not solve this problem – you may call such the associated names “validly published” but this does not solve the real-world issue. Other researchers could still not replicate the research because they could not access the cultures. Only more science-friendly legislation will solve this issue. Everything else is just relabeling or whitewashing.

4 July – Markus Göker

As for “what makes using DNA is that it democratises / increases the ability of microbiologists globally to participate. – all you need is an ability to extract DNA and online access. This is fantastic - the more people are engaged, the more microbiology is advanced! So given the above, what would the researcher community (some with potentially limited means) choose?” you fail to mention in exactly what “microbiologists globally” can now “participate”. First of all, those who do not have the resource for genome sequencing or metagenomics still cannot participate in this. They may well want to rely on cheaper cultivation-based methods. Apart from that, what does the fact that people want to do metagenomics or other genome sequencing even have to do with the entire question we are discussing here? Contrary to what publications in favor of the 2016 Whitman proposal or of the SeqCode have been claimed, the ICNP has never hindered anybody to do genome

sequencing, to propose a name for a prokaryote based on a genome sequence, or to describe a taxon based on a genome sequence. Nobody was ever hindered to participate in any of this. The BOBW authors have clarified this many times; I'm just not sure whether you ever read any of their publications or listened to any of their talks.

4 July – Markus Göker

I may quote here an e-mail message I've received back in March from Prof. Bharat Patel:

"Dear Markus,

I retired in 2017 after a career in Systematics, taxonomy and ecology of thermophiles from volcanic and deep geothermal aquifers. However, I have held my interest in this and have been following the debate on SeqCode. I was very concerned about the use of "abstract sequence data as the main driver of biological understanding". SeqCode can be an integrated part but not the driver form may reasons. However, I felt that the debate should be best left to the next generation of researchers.

I was very pleased to see the very balanced article presented by you and your colleagues. Developing tools for bioinformatics, their use and their use in product development are different areas of specialization and could be harmonized. If SeqCode was made the sole tool for S&T, it would cause major issue to the microbiologist who use bioinformatics as mere tools. The diversity extant is now al but know, understanding function and improving function of microbiomes is important which can be better understood by using technologies that are currently available to us rather creating more confusion.

Microbiology, the ability to grow microbes, has been a skill that has almost disappeared from the advanced economies. It has been replaced by molecular biology, bioinformatics and biotechnology which are either technologies or products. Students cannot differentiate the meaning of these "buzz" words and careers have been built entirely on a single gene or technique ate expense of a more holistic biology. Hopefully, a more inclusive scenario can be discussed and developed.

Congratulations on the very balanced views on the current debate / discussion.

Kind regards and best wishes.

Bharat"

4 July – Markus Göker

As for "The ICSP has an obligation to provide the best system that provides utility to a majority of whom we represent", the funny thing is that in the sentence before you are only referring to those who propose names, which is not the "majority of whom we represent". The ICSP is not a lobbying organization for taxonomists. This a common mistake that has been highlighted in the last plenary meeting. The ICSP has no obligation to make life easier at all cost for those who propose names. There are also many other people (and these form the majority). The "best system" is an informative and fair system, with a sufficient incentive both for naming prokaryotes based on genome sequence and for cultivating and depositing even the same prokaryotes later. Believe it or not, this is what BOBW provides.

4 July – Markus Göker

As for "The option where your nomenclature choice can be superseded because a priority ruling (i.e. the issues around rule 72(4)), or, having it recognised by the SeqCode as having equivalent status?", as I have mentioned before, you might want to look up the meaning of priority as used in the ICNP. For instance, a code of nomenclature cannot "grant priority"; this is in conflict with how "priority" is defined in the code.

4 July – Markus Göker

As for "The option where your nomenclature choice can be superseded because a priority ruling (i.e. the issues around rule 72(4)), or, having it recognised by the SeqCode as having equivalent status? The reality I suspect is most will go for the later or opt for neither and go with the wildwest status quo. If this is the case, then are we, as a community, in a better position?", please just consider what the BOBW authors have already said several times about 72(4) (including on BISMiS Live here in this forum) and please see the list of 4 future options at the end of my BISMiS Live talk (was also given in the ICSP plenary).

4 July – Markus Göker

As for "it would good to see how the authors intend on addressing issues around rule 72(4) and their proposal around genome quality minimal standards", please see the comments already made here and see the BISMiS Live talk (was also given in the ICSP plenary). Also note that there are people who do cultivate and deposit the first representative of, say, a phylum, and that their interest may well be that the phylum is then named after their genus and not named after a just a MAG. Fairness does not mean treating everyone equally. It means treating the same situation equally. If you give one product to one customer for 20 Dollar and 5 minutes later the same product to another customer for 10 Dollar, you have treated the two customers equally (you gave them the same thing). But you have not treated them fairly. The first of the two customers may indeed get very upset about this.

5 July - Markus Göker

To ease navigation through the BISMiS Live session with the BOBW authors, here are links directly to the sections:

Introduction: https://youtu.be/7HzzoKZMsgw

Ed Moore's talk: https://youtu.be/7HzzoKZMsgw?t=264

Henrik Christensen's talk: https://youtu.be/7HzzoKZMsgw?t=1314

Markus Göker's talk: https://youtu.be/7HzzoKZMsgw?t=2255

Discussion: https://youtu.be/7HzzoKZMsgw?t=4700

6 July – Aharon Oren

The six-month discussion on the Best of Both Worlds paper is now closed. According to Article 13 (b) (4) of the statutes of the ICSP, the authors may respond to the comments in the coming two months. I will send out the ballot to the voting members of the ICSP on September 6 or earlier, in case the authors of the proposal will notify me that they do not wish to post any further responses. The collated comments and responses posted on Slack will be added to the ballot material.
